# Targeting ryanodine receptors to treat human diseases

**DOI:** 10.1172/JCI162891

**Published:** 2023-01-17

**Authors:** Andrew R. Marks

**Affiliations:** Department of Physiology and Cellular Biophysics, Columbia University Vagelos College of Physicians and Surgeons, New York, New York, USA.

## Abstract

This Review provides an update on ryanodine receptors (RyRs) and their role in human diseases of heart, muscle, and brain. Calcium (Ca^2+^) is a requisite second messenger in all living organisms. From *C. elegans* to mammals, Ca^2+^ is necessary for locomotion, bodily functions, and neural activity. However, too much of a good thing can be bad. Intracellular Ca^2+^ overload can result in loss of function and death. Intracellular Ca^2+^ release channels evolved to safely provide large, rapid Ca^2+^ signals without exposure to toxic extracellular Ca^2+^. RyRs are intracellular Ca^2+^ release channels present throughout the zoosphere. Over the past 35 years, our knowledge of RyRs has advanced to the level of atomic-resolution structures revealing their role in the mechanisms underlying the pathogenesis of human disorders of heart, muscle, and brain. Stress-induced RyR-mediated intracellular Ca^2+^ leak in the heart can promote heart failure and cardiac arrhythmias. In skeletal muscle, RyR1 leak contributes to muscle weakness in inherited myopathies, to age-related loss of muscle function and cancer-associated muscle weakness, and to impaired muscle function in muscular dystrophies, including Duchenne. In the brain, leaky RyR channels contribute to cognitive dysfunction in Alzheimer’s disease, posttraumatic stress disorder, and Huntington’s disease. Novel therapeutics targeting dysfunctional RyRs are showing promise.

## Introduction

**Figure 1:**
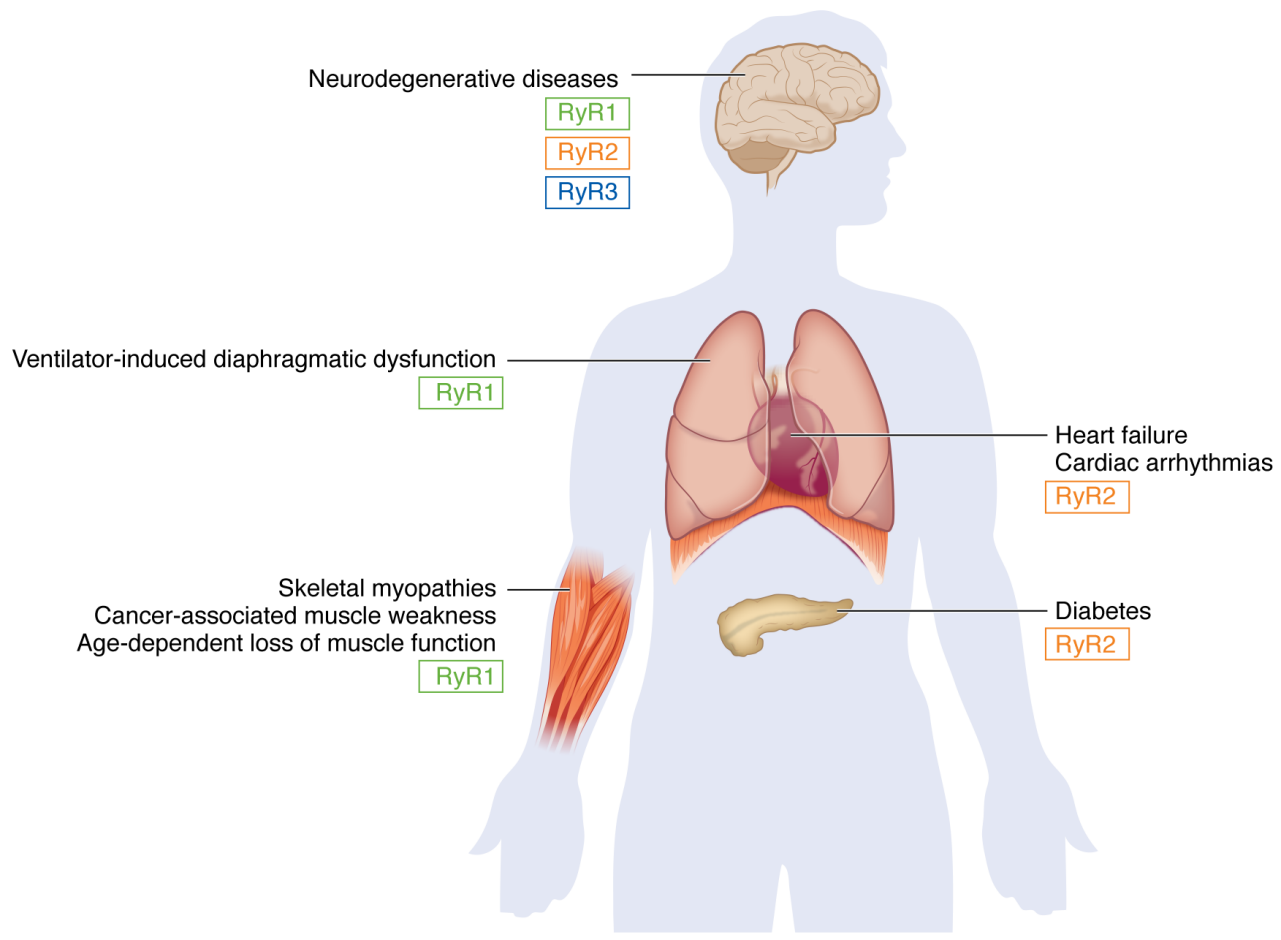
Leaky ryanodine receptors are involved in normal and pathological physiology in multiple organs. The figure indicates links of ryanodine receptors RyR1–RyR3 to neurodegenerative diseases (4, 26–28, 52, 84, 85, 88), ventilator-induced diaphragmatic dysfunction (81–83), heart failure (2, 7, 8, 46, 48, 53, 58, 65, 65–70, 75), cardiac arrhythmias (3–6, 11–19, 64, 66), skeletal myopathies (22–24, 49, 80, 87, 89), cancer-associated muscle weakness (50), age-dependent loss of muscle function (51), and diabetes (29). Adapted with permission from *Biochimica et Biophysica Acta: Molecular Cell Research* (55).

Ryanodine receptors (RyRs), homotetrameric intracellular Ca^2+^ release channels composed of four 565 kDa (~5,000 amino acids) protomers, are found on the sarcoplasmic/endoplasmic reticulum (SR/ER) of virtually all cell types (1). They are so named because they bind ryanodine, an extract of the *Ryania speciosa* plant used on blow darts by indigenous peoples in South and Central America to paralyze prey. Ryanodine locks RyR channels in an open state resulting in uncontrolled release of SR Ca^2+^ and muscle tetany. RyR dysfunction is linked to clinical disease across a wide range of disorders (Figure 1).

### RyR dysfunction in heart failure.

Chronic diastolic SR Ca^2+^ leak due to stress-induced remodeling (caused by PKA phosphorylation and oxidation) of RyR2 (cardiac muscle) and RyR1 (skeletal muscle) channels depletes SR Ca^2+^, reduces contractility, promotes heart failure (HF) progression (2), is arrhythmogenic (3–6), and impairs skeletal muscle function (7). A new class of small-molecule drugs known as Rycals fix RyR1 and RyR2 leak by preventing the stress-induced depletion of the stabilizing subunits calstabins (also known as FKBPs) (8) from the RyR macromolecular complexes (2, 9–11) and improve myocardial and skeletal muscle performance in mice.

### RyR dysfunction in arrhythmias.

SR Ca^2+^ leak through RyR2 can cause arrhythmias due to delayed after-depolarizations (DADs) of the cell membrane driven by activity of the electrogenic Na^+^/Ca^2+^ exchanger NCX (6, 12, 13). Chronic PKA phosphorylation of RyR2 at Ser2808 leading to calstabin2 (also known as FKBP12.6) depletion and diastolic SR Ca^2+^ leak was linked to ventricular arrhythmias (3). RyR2s are leaky in atrial tissue from humans and dogs with chronic atrial fibrillation (AF) (14), and calstabin2-deficient mice exhibit AF (15).

**Figure 2:**
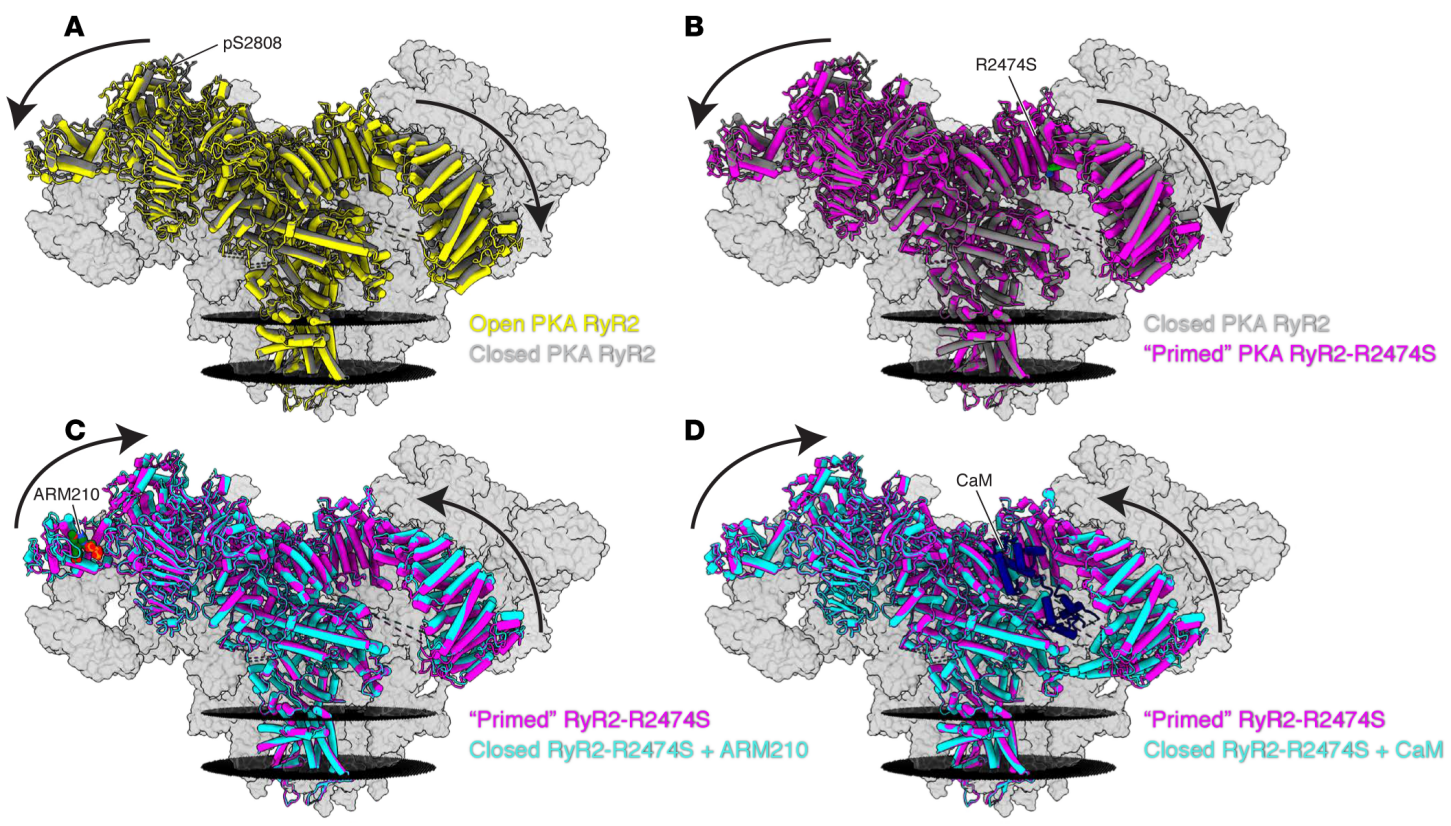
Cryo-EM reconstructions of human RyR2. The reconstructions show that the CPVT mutant RyR2-R2474S puts the channel into a primed state partway between closed and open, and treatment with the Rycal ARM210 or calmodulin puts the channel back toward the closed state, preventing leak. (**A**) Models of open PKA-phosphorylated RyR2 (Protein Data Bank [PDB; https://www.rcsb.org/]: 7U9R; yellow) and closed PKA-phosphorylated RyR2 (PDB: 7U9Q; gray). Arrows show the cytosolic shell of the PKA-phosphorylated RyR2 shifting downward and outward when the channel goes from the closed to the open state. Only the front protomer is colored. The sarcoplasmic reticular membranes are black discs. Conditions include 10 mM ATP, 150 nM free Ca^2+^, and 500 mM xanthine. (**B**) Models of closed PKA-phosphorylated RyR2 (PDB: 7U9Q; gray) and primed PKA-phosphorylated RyR2-R2474S (PDB: 7U9X; magenta), with arrows showing the cytosolic shell of RyR2-R2474S shifting downward and outward compared with closed PKA-phosphorylated RyR2, similar to the structural changes observed for PKA-phosphorylated RyR2 when the channel goes from closed to open. We define this state between closed and open as the primed state. (**C**) Models of primed PKA-phosphorylated RyR2-R2474S (PDB: 7U9X; magenta) and closed PKA-phosphorylated RyR2-R2474S + ARM210 (PDB: 7UA1; cyan). Arrows show the cytosolic shell of PKA-phosphorylated RyR2-R2474S + ARM210 shifting upward and inward compared with RyR2-R2474S, reversing the primed state closer to the closed state. (**D**) Models of primed PKA-phosphorylated RyR2-R2474S (PDB: 7U9X; magenta) and closed PKA-phosphorylated RyR2-R2474S + calmodulin (CaM) (PDB: 7UA3; cyan). Similarly to the Rycal ARM210, CaM reverses the primed state back to the closed state. Reconstructions were adapted with permission from *Structure* (74).

Catecholaminergic polymorphic ventricular tachycardia (CPVT) is an inherited disorder characterized by ventricular arrhythmias during exercise in a structurally normal heart (3, 16). Mutations in RyR2 are responsible for most cases of CPVT (denoted as CPVT-1) (3, 16), while mutations in calsequestrin (CASQ) are responsible for an even rarer autosomal recessive form of the disease (CPVT-2) (17). Depletion of calstabin2 from the channel results in increased sensitivity to cytosolic Ca^2+^, aberrant diastolic SR Ca^2+^ release, DADs, and fatal arrhythmias (3). Heterozygous CPVT mutant RyR2-R2474S mice exhibit exercise-induced ventricular arrhythmias, and sudden cardiac death, which can be prevented by treatment of the mice with Rycals (3, 11, 16, 18). We recently solved the structure of RyR2-R2474S and showed that it is in a primed state (Figure 2) that renders it more easily activated, accounting for the diastolic SR Ca^2+^ leak that triggers the fatal arrhythmias (19).

### RyR dysfunction in malignant hyperthermia.

Malignant hyperthermia (MH) is linked to RyR1 mutations. Patients develop a hypermetabolic response (hyperthermia, acidosis, and rhabdomyolysis) following exposure to volatile anesthetic gases including halothane and isoflurane as well as the muscle relaxant succinylcholine (20). The therapy for MH is immediate administration of dantrolene (21).

### RyR1-related myopathies.

Mutations in RyR1 cause a rare form of congenital myopathies collectively known as RyR1-related myopathies (RyR1-RM) (22). Other congenital myopathies, including Duchenne muscular dystrophy and limb-girdle muscular dystrophy (β-sarcoglycan deficiency), have also been linked to RyR1 dysfunction and Ca^2+^ release (23, 24). Antibodies against RyR1 are present in some patients with myasthenia gravis, an autoimmune disease characterized by recurrent episodes of muscle weakness (25).

### RyR dysfunction in cognitive dysfunction.

Posttraumatic stress disorder (PTSD) afflicts many soldiers returning from Iraq and Afghanistan, and there is no current effective therapy. In a murine PTSD model, RyR2 channels were leaky, and Rycal-treated mice (26) and RyR2-S2808A mice were protected against cognitive and behavioral dysfunction (26). In murine models of Alzheimer’s disease, neuronal RyR2 channels were leaky, and treatment with the Rycal S107 reduced amyloid production and improved cognitive and behavioral function in mice (27, 28).

### RyR dysfunction in metabolic disorders.

CPVT mouse models expressing leaky RyR2 exhibit impaired oral glucose tolerance. Pancreatic β cells from CPVT mice have decreased ER Ca^2+^ stores as a result of RyR2 Ca^2+^ leak. This leads to mitochondrial dysfunction, impaired cellular energetics, reduced ATP production, and defective activation of the K_ATP_ channel that is involved in signaling for insulin secretion (29). Treating diabetic mice with S107 to inhibit the RyR2-mediated ER Ca^2+^ leak improves mitochondrial function, and insulin secretion (29). We found that patients with CPVT-linked RyR2 mutations have reduced glucose tolerance and decreased insulin levels exactly as seen in the murine models (29).

In this Review, the evolution of our understanding of RyR channels is presented; insights regarding mechanisms of human diseases linked to RyR dysfunction and their treatment gained from studying RyR, in particular using cryogenic electron microscopy (cryo-EM) to solve its structure, are discussed; and directions for future research on RyR are proposed.

### RyR: structure and function — from cloning to cryo-EM.

**Figure 3:**
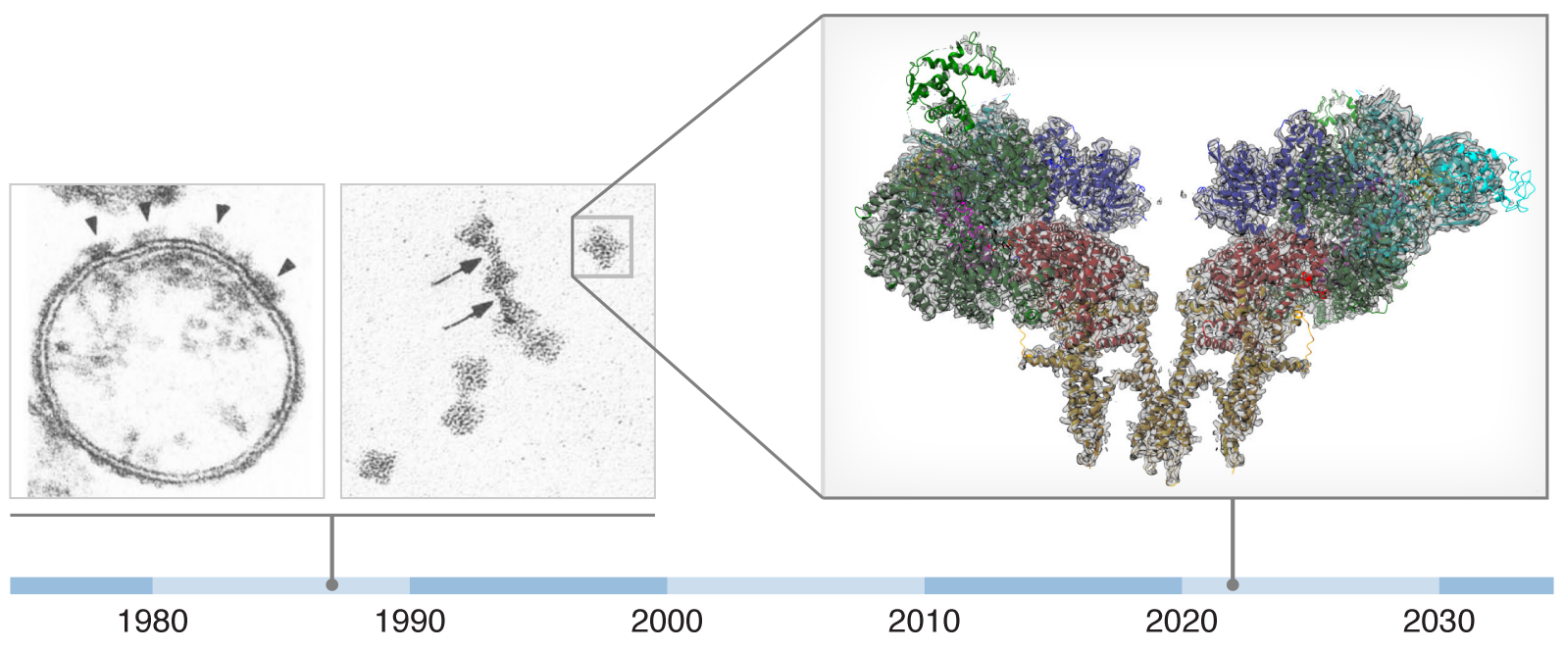
Evolution of knowledge about the ryanodine receptor from initial purification to function to structure. Left: Negative stain electron microscopic images of the “feet structures” of junctional terminal cisternae vesicles and purified ryanodine receptors first visualized in 1987 (images reproduced with permission from the *Journal of Biological Chemistry*; ref. 31). By comparing the shape and size of the purified RyR to that of the densities protruding from the surface of the SR vesicles, Fleischer and colleagues concluded that RyR was the foot structure spanning the gap between the terminal cisternae of the SR and T-tubules. But it was not known until later when RyR1 was cloned and functionally expressed that it was indeed the Ca^2+^ release channel required for muscle excitation-contraction coupling (37). Arrowheads denote individual RyR1 channels in the SR membrane; arrows denote purified, isolated individual RyR1 channels. Original magnification, ×140,000. Right: The cryo-EM structure of RyR1 at approximately 2.4 Å resolution; two opposing protomers are shown in the side view (adapted with permission from *Structure*; ref. 74).

RyR was first described by Clara Franzini-Armstrong and colleagues (30) and Sidney Fleischer and colleagues (31) as the “foot structure” (Figure 3) spanning the gap between the terminal cisternae of the SR and the T-tubule based on negative staining electron micrographs. The identification of these anatomic structures with the RyR protein was facilitated by purification using the high-affinity ligand ryanodine.

Before it was known to be a Ca^2+^ channel, Fleischer had partially purified RyR using tritiated ryanodine binding and sucrose gradients. The result was three bands on a Coomassie-stained gel with approximate molecular weights of 300, 170, and 100 kDa, and he thought the channel was a trimeric complex. Later we determined that these three bands were proteolytic fragments of the 565 kDa type 1 RyR (RyR1) monomer, four of which constitute the homotetrameric channel.

Collaborating with Paul Tempst when we were both at Harvard, I managed to obtain the sequences of RyR1 peptides (32) and used these to generate probes to screen cDNA libraries and clone the RyR1 cDNA (33) at roughly the same time as Numa (34) and MacLennan (35). In those days before PCR and automated sequencing, cloning the RyR1 cDNA was something of a feat. In fact it wasn’t until the first RyR1 Northern blot that I understood the size of its mRNA (33) — a massive 16 kb!

The next challenge was to try and figure out the function of the protein encoded by this massive mRNA. To achieve this required pushing the envelope of available technology, and I decided to express the channel in Sf9 insect cells, in part because they lacked the stabilizing subunit FKBP12 (which I renamed calstabin1 once I discovered its natural cellular function as the calcium channel–stabilizing binding protein) and because this system was capable of expressing high levels of large proteins. I stumbled upon calstabin1 as a subunit of the RyR1 macromolecular complex (36) as a result of the peptide sequencing experiment when its amino terminus showed up in the sequences (32). It turned out that this peptidyl-prolyl isomerase was a component of the RyR1 channel macromolecular complex that we later went on to describe in greater detail (2, 9, 10).

The Sf9 insect cell system required cloning the RyR1 16-kb cDNA into a transfer plasmid that was then used to recombine with viral DNA and infect the insect cells that produced the protein of interest. Because the RyR1 cDNA was so large, it carried with it a growth disadvantage and was being clipped out with only truncated proteins being expressed. Once I eliminated the early amplification step, this problem was solved, and we were able to isolate full-length RyR1 protein, reconstitute it into lipid liposomes fused to planar lipid bilayers in a recording chamber, and show that the protein encoded a Ca^2+^ channel (37). This experiment allowed us to determine the functional role of calstabin1 in the RyR1 complex as well: it stabilized the closed state of the channel (37). We subsequently cloned the RyR2 isoform from heart (3), as well as several closely related channels, the type 1 and 2 inositol 1,4,5-trisphosphate receptors IP3R1 (38) and IP3R2 (39). All revealed high degrees of homology, indicating evolution from a common ancestral cation release channel closer to IP3R channels that acquired more complexities encoded by the much larger RyR channel genes (40).

While defining the regulation of the RyR channels, I became interested in signaling mediated by their phosphorylation and oxidation, both of which occur in disease states such as HF, skeletal myopathies, and neurodegenerative disorders. This interest led to the definition of the RyR1 and RyR2 macromolecular complexes, composed of kinases, phosphatases, phosphodiesterases, and their targeting proteins as well as calstabins (2, 10) that regulate the release of intracellular Ca^2+^ from ER/SR in response to stress.

After more than two decades working on RyRs, I was tremendously excited to finally see what they really looked like at near-atomic resolution. Collaborating with Wayne Hendrickson and Joachim Frank, our group, along with two others (41, 42), was able to solve the structure of the mammalian RyR1 using cryo-EM (43). First at 4.8 Å overall resolution and later at better than 2.5 Å resolution, we were able to see almost the entire structure of the channel and identify ligand and drug binding sites (44). The beautiful models revealed a cation channel comprising four subunits, each with six transmembrane segments but without a voltage sensor, that was otherwise structurally homologous to voltage-gated ion channels (43). The unique and enormous cytoplasmic domain promised to hold a treasure trove of findings that would keep us occupied for years to come. It had taken approximately 30 years to progress from the “foot structure” images (Figure 3) to the near-atomic-resolution models (Figure 2).

## Discovering the RyR leak

Working with Robert (“Rocky”) Kass and Jon Lederer on understanding the molecular basis for cardiac arrhythmias led to my interest in how sympathetic nervous system activation linked to stress affected RyR2 channels in the heart. I contacted Bob Lefkowitz, the guru of adrenergic receptors and my father’s former student when he was at Columbia (45), and obtained one of his transgenic mouse models overexpressing β-adrenergic receptors. RyR2 channels in the hearts of these mice were massively PKA phosphorylated and highly active (2). I reasoned that since heart failure was well known to be a chronic hyperadrenergic state and was highly associated with cardiac arrhythmias, there might also be PKA phosphorylation of RyR2 channels in failing hearts. So we looked at human hearts from cardiac transplant patients and animal models of HF and discovered that indeed RyR2 channels were heavily PKA phosphorylated in HF. The net result of this PKA phosphorylation was to decrease the binding of the stabilizing subunit, calstabin2, rendering the channels leaky to Ca^2+^. This SR Ca^2+^ leak via calstabin2-depleted highly phosphorylated RyR2 channels had two major adverse consequences: (a) depletion of the SR Ca^2+^ store, directly impairing contractility (46); and (b) aberrant release of Ca^2+^ during diastole, causing delayed after-depolarizations (DADs) that trigger fatal cardiac arrhythmias (3). Later we figured out that the HF channels were not only PKA phosphorylated but also oxidized and nitrosylated, all of which combined to reduce calstabin2 binding and promote SR Ca^2+^ leak (47, 48).

Following the discovery of the leaky RyR2 in failing hearts, we went on to show similar stress-induced leak in skeletal muscle affecting the RyR1 isoform during extreme exercise (49), and also in Duchenne muscular dystrophy (DMD) (24), cancer-associated loss of muscle function (50), age-dependent loss of muscle function (51), and RyR1-RM (22). In addition, we showed that mutant RyR2 channels linked to exercise-induced sudden cardiac death or CPVT (3, 16) were also leaky. We found that murine models of human CPVT with mutant leaky RyR2 also quite unexpectedly displayed glucose intolerance secondary to impaired insulin secretion due to leaky RyR2 channels in their pancreatic β cells (29). This surprising finding was supported when we examined CPVT patients harboring mutant leaky RyR2 channels and found them to be glucose intolerant with reduced insulin levels (29).

Cognizant of the long history of a possible role for Ca^2+^ abnormalities in Alzheimer’s disease, we further looked into that disorder, as well as PTSD, and found roles for leaky RyR2 channels in both (26, 28); more recently, we found possible involvement in the brain fog associated with long COVID (52).

## Fixing the RyR leak

The question remained: what could be done to fix leaky RyR2 channels? Based on the observation that leak was associated with a reduction of calstabin binding to the channels, I looked for a drug that could prevent this loss. A Japanese group at Yamaguchi University had closely followed the work from my laboratory (2) and was testing various drugs to see whether any could fix the leak in a canine model of HF when they stumbled upon JTV-519 (53). This was a 1,4-benzothiazepine synthesized by Japan Tobacco Co. that was a multichannel blocker but also bound to RyR channels and prevented loss of calstabin2 from RyR2, fixed the leak, and improved function in HF animals. Since JTV-519 was a “dirty” drug (i.e., a blocker of multiple ion channels, including the HERG potassium channel) and pretty much insoluble, we started a company, ARMGO Pharma Inc., to develop better drugs we called Rycals that would be more specific for RyR channels and with better pharmaceutical properties (11). Rycals proved effective in reducing HF progression and cardiac arrhythmias and improved muscle function in animal models and in an early clinical trial in HF patients run with the French pharmaceutical company Servier. A second-generation Rycal (ARM210) recently completed a phase Ib study at the NIH with promising results showing improved muscle strength in RyR1-RM patients and no safety concerns. A more definitive Rycal clinical trial in RyR1-RM patients is being planned, and a trial in patients with CPVT will likely start this year at the Mayo Clinic and in Amsterdam.

## Opposing views: seeking common ground through structure

Some of our findings have been challenged by several groups in the field, and these differences have been addressed in some detail previously (54, 55). These groups have questioned whether Ser2808 is the only or even the major site of PKA phosphorylation in RyR2, and whether its phosphorylation plays a role in adrenergic regulation of the channel and in HF. We detected robust PKA phosphorylation of Ser2808 by mass spectrometry; we did not observe PKA phosphorylation at Ser2030 (19). However, a mouse in which the RyR2-S2030 site was mutated to alanine was reported to exhibit defective responses to β-adrenergic stimulation (56).

In agreement with our studies, an independently generated RyR2-S2808A mouse clearly showed significant blunting of the isoproterenol-stimulated Ca^2+^ transient and cell shortening (see Figure 5, C and E, in ref. 57) in cardiomyocytes compared with wild-type controls when stimulated at 3 Hz. Importantly, strong support for our findings comes from an independently generated RyR2-S2808A mouse that also showed complete preservation of cardiac function in comparison with wild-type mice (see Supplemental Table 1 in ref. 57 [fractional shortening, second line from the bottom]) following transverse aortic constriction (TAC), which induced cardiac dysfunction in wild-type mice but not in the RyR2-S2808A mice. Indeed, this group found no loss of function in the RyR2-S2808A mice 11 weeks after TAC compared with a significant loss of function in WT controls (see Supplemental Table 1 in ref. 57). Thus, our RyR2-S2808A mice and those from another group both exhibited blunted responses to catecholamines and are protected against HF progression (47, 48, 58). While the confirmatory papers from an independent laboratory have been cited as evidence against our findings rather than supporting them (57, 59, 60), the fact is that in all these studies, the RyR2-S2808A mice had normal heart dimensions before and after banding and thus were protected against HF. Valdivia and colleagues generated a second RyR2-S2808A mouse in the same genetic background as ours (C57BL/6) and reported a lack of protection against HF at odds with both their own previous study (57) and ours (58).

The role of calstabins as stabilizing subunits of RyR channels has also been disputed (61). We originally found that calstabin1 (FKBP12) is a subunit of RyR1 (36) and that it stabilizes the channel closed state, thereby preventing aberrant SR Ca^2+^ leak (37). Calstabin2 (FKBP12.6), which is a subunit of RyR2 (62, 63), plays an important physiological role in modulating RyR2 function (64–71). However, Bers and colleagues reported that calstabin2 plays no role in cardiac physiology (72), in contrast to a previous finding from his own laboratory that was described as being “consistent with an important SR-stabilizing effect of FKBP in intact rat ventricular myocytes” (73).

As noted below, structural studies are now revealing further insight into the precise mechanism by which calstabins bind to and stabilize RyR1 and RyR2. These studies and others that are forthcoming should help settle the differences in this field.

## Current understandings and looking to the future

### Novel therapeutics target leaky RyR channels.

With the above brief background, it should be clear that important unanswered questions must be addressed to better understand how RyR channels become leaky and how Rycal drugs fix the leak. Toward this end, we have recently solved the high-resolution structures of RyR1 and RyR2 bound to the Rycal ARM210, identifying the binding site for the drug (19, 74). Unexpectedly, the Rycal binding site is in a cleft at the periphery of the channel that also binds ATP or ADP (74). The rings of the Rycal form π stacks with tryptophan residues in the binding site and with the adenine ring of the ATP/ADP. Addition of ATP to the Rycal in a binding reaction increases the B_max_ of the reaction approximately 10-fold (because ATP is the binding partner for the Rycal in the RyR binding site) (19). Since the drug binding site is about as far from the channel pore as possible, it raises the question of how drug binding all the way out in the periphery of the cytoplasmic domain can fix a leak in the channel pore.

To address this question, we first solved structures of the CPVT-linked RyR2-R2474S mutant channel, in part because prior observations told us that the Rycal drugs do not bind well to non-modified (i.e., non-PKA-phosphorylated, non-oxidized) wild-type channels (19). Moreover, we wanted to understand how mutations in the cytoplasmic domain of the channel could render the channel leaky. Bingo — solving the structure of RyR2-R2474S with and without ARM210 yielded answers to both questions (19).

First, we found that the mutant RyR2-R2474S channel linked to CPVT had an overall alteration in its structure as determined by cryo-EM (19). We expressed the human recombinant RyR2 in HEK293T cells and purified it for cryo-EM; this allowed us to also introduce the CPVT mutations into the channel. The entire CPVT channel structure had been shifted to what we defined as a “primed state” — essentially halfway between open and closed (19). This suggested that the channel could be more readily activated by Ca^2+^, which would explain why the channel was unstable and leaky under conditions in which a normal channel was tightly closed (e.g., during diastole when the resting [Ca^2+^]_cyt_ is very low, ~100 nM, and normal RyR channels are not active). We were able to make this determination because of the remarkably high resolution we had achieved — approximately 2.5 Å for the entire channel and even higher in some regions (19).

### RyR posttranslational modifications, channel structure, and HF.

I have previously noted the parallelism between CPVT-causing RyR2 mutations that result in SR Ca^2+^ leak and the leak present in otherwise normal RyR2 channels in failing hearts (46, 75, 76, 86). The former are due to a mutation in the channel that we now know places the channel into the primed state (19), and the latter is due to posttranslational modifications that displace the stabilizing subunit calstabin2 and cause SR Ca^2+^ leak. This observation led naturally to the question of whether the posttranslational modifications themselves affected the overall structure of the channel. To address this question, we solved the structure of the PKA-phosphorylated RyR2 channel to mimic one of the common posttranslational modifications observed in HF RyR2 channels (19).

Remarkably, PKA phosphorylation of the channel, which occurred primarily on Ser2808 as determined by mass spectrometry analyses, also placed the channel into a primed state (19). However, compared with the primed state of the CPVT mutant RyR2-R2474S channel, the PKA phosphorylation–induced primed state was less open (19). This makes sense because the CPVT mutation is a pathological state that causes exercise-induced sudden cardiac death, a catastrophic outcome. In contrast, PKA phosphorylation of the channel occurs with normal exercise (49) and is not necessarily lethal, although when the channel becomes chronically PKA phosphorylated, as is the case in HF, the resulting chronic SR Ca^2+^ leak depletes the SR Ca^2+^, contributes to impaired contractility, and triggers arrhythmias, which are common in HF (2).

Another unresolved question that had been haunting us for years was how the CPVT mutations and posttranslational modifications could cause reduced binding of calstabin2 to the channel. The answer was hidden in a paper we had published in *Cell* back in 2003, before anything was known about the RyR2 structure and the primed state had not yet been conceived. Figure 4D in this paper showed that the binding affinity of calstabin2 to three different CPVT mutant channels was reduced (3)! This experiment showed that the primed state, which these CPVT channels were in due to the mutations in RyR2 (19), had a reduced affinity for the stabilizing subunit calstabin2, which fit beautifully with our current structural data. This would explain why the mutant CPVT channels were leaky: they had reduced binding to the stabilizing subunit and were in a primed state such that they were more readily activated at low (normally non-activating) [Ca^2+^]_cyt_.

The next question is what happens in HF. Are RyR2 channels that are oxidized and phosphorylated in failing hearts also in a primed state? If so, does this explain why they are depleted of calstabin2 and leaky? These are good questions and will require additional studies to firmly address. For example, we need to be able to purify sufficient amounts of RyR2 from failing hearts to solve its structure and in so doing preserve the “native” condition of the channel. Another challenge is to figure out whether and how the primed state affects the binding pocket for calstabin2 in the channel. To solve this question, we have had to purify the channel without calstabin2 bound, and structures of these channels should be published soon.

Next we asked: how does Rycal binding to the channel fix the leak? Recall that the Rycal binding site is far away from the pore of the channel. Again the structures provided an unexpected answer. Binding of the Rycal ARM210 shifted the mutant CPVT RyR2-R2474S structure back from the primed state toward the stable closed state of the channel — fixing the leak without impairing the ability of the channel to open normally (19).

### Controversy over RyR’s PKA phosphorylation site.

The PKA phosphorylation site at Ser2808 has become something of a lightning rod in the field, attracting some attention and controversy as noted earlier. In our mass spectrometry analyses (19), Ser2808 was PKA phosphorylated at least an order of magnitude more abundantly than any other site in the channel. Yet there are certainly other sites in RyR2 that can be PKA phosphorylated in vitro (77). There is also the question of the relative role of CaMKII phosphorylation of RyR2. It has been proposed that CaMKII phosphorylation of RyR2 at S2814 and not PKA phosphorylation at S2808 causes SR Ca^2+^ leak (78). We generated knockin mice with alanine or aspartic acid substitutions for Ser2808 (PKA site) and the nearby Ser2814 (CaMKII site). In our hands, substitution of an alanine for Ser2808 (the RyR2-S2808A mouse) ablated PKA phosphorylation of the channel and severely blunted the effects of isoproterenol in terms of increasing contractility and heart rate, whereas alanine substitution for Ser2814 did not. In fact, alanine substitution for Ser2814 resulted in a mouse that was defective in the Bowditch phenomenon, otherwise known as the positive staircase in which increasing heart rate results in increasing contractility (79). We reasoned that this showed that the Ser2814 site was a sensor for global [Ca^2+^]_cyt_ and that, as heart rate increased, the integral of [Ca^2+^]_cyt_ increased, and this in turn would activate CaMKII, leading to phosphorylation of Ser2814 and activation of RyR2 to release more SR Ca^2+^ to ensure that contractility increased to avoid a loss of output at higher heart rates.

### RyR-targeting drugs in HF and myopathy trials.

The future holds clinical trials; two are currently planned and others are being considered. This year a two-center trial will be conducted at the Mayo Clinic and in Amsterdam testing the Rycal ARM210 in patients with CPVT and known RyR2 mutations. This trial will include some form of exercise testing and ambulatory arrhythmia monitoring in patients who have residual arrhythmia on standard therapy. The potential benefit of a Rycal in this setting is that it could be disease modifying, avoid the adverse effects of beta blockade, and not require titration to heart rate and blood pressure since it has little effect on either.

A second trial will potentially provide definitive answers as to whether a Rycal (ARM210) is safe and beneficial for patients with RyR1-RM. RyR1-RM is, in some patients, a progressive myopathy that results in loss of muscle function over time. There is no current treatment. More severely affected patients typically have recessive genotypes, and more moderately affected individuals are typically those with dominant forms of inheritance. Again, a Rycal could be disease modifying in these patients. Another muscle disorder for which a Rycal could be beneficial is DMD. In DMD, RyR1 channels in skeletal muscle are leaky because they become nitrosylated as a result of an upregulation of the inducible form of nitric oxide synthase (iNOS) (24). The nitrosylation decreases the binding affinity of calstabin1 for the channel (24), possibly because it places the channels in a primed state, but we need structural data to ascertain this. We initially showed efficacy for Rycals in DMD using the *mdx* mouse model (24), and these findings were independently confirmed by two other groups (80). If a Rycal is well tolerated and beneficial in DMD, it could be transformative therapy, because the major cause of mortality in boys with DMD is heart disease as the boys develop a cardiomyopathy and arrhythmias. A Rycal could potentially treat the skeletal muscle and cardiac myopathies and the arrhythmias. Moreover, another major symptom in boys with DMD is pulmonary failure due to impaired respiratory function caused by diaphragmatic weakness, and Rycals improve diaphragm function in animal models (81–83).

### Targeting RyR in other diseases.

In another study, we found that murine models of human CPVT exhibited glucose intolerance and impaired insulin secretion (29). This was because leaky RyR2 channels in pancreatic β cells caused ER stress and mitochondrial dysfunction leading to reduced ATP production, decreased K_ATP_ channel activity, and reduced voltage-dependent Ca^2+^ influx, which reduces insulin release. Treatment of murine models of diabetes mellitus (DM) improved insulin release and glucose tolerance (29). A Rycal could be an excellent therapeutic in DM because it wouldn’t be limited by the potential for hypoglycemia like many diabetes therapeutics. Moreover, since most DM patients also have heart disease, it could treat that as well, whereas many current diabetes therapeutics exacerbate heart disease.

Cancer-associated muscle weakness is the major paraneoplastic syndrome suffered by breast cancer and other patients. Together with Theresa Guise we used a murine model of human breast cancer metastatic to bone to show that TGF-β released from the bone activated a pathway leading to oxidation of RyR1 in the neighboring skeletal muscle, rendering the channels leaky and promoting muscle weakness (50). Either fixing the leak with a Rycal or preventing bone resorption with an osteoclast inhibitor could reduce the leak and improve muscle strength in mice (50).

Age-dependent loss of muscle function, also known as “sarcopenia” (importantly, this term describes only the loss of muscle mass, not the loss of intrinsic muscle function), is a leading cause of morbidity and mortality in aging. Using aged mice from a colony maintained at the National Institute on Aging, we showed that progressive oxidative overload associated with normal aging could cause RyR1 channel leak and contribute to reduced muscle function (51). Fixing the leak with a Rycal improved muscle function in aged mice (51).

The brain is a particularly challenging organ to treat, and neurodegenerative disease has reached epidemic proportions while evading effective therapy. We first became interested in neurological diseases for the simple reason that the identical RyR1 and RyR2 channels present in skeletal and cardiac muscles, respectively, are also expressed in different regions of the brain. It made sense therefore to see whether stress could induce leaky RyR channels in the brain. We looked at a murine model of PTSD, which afflicted thousands of soldiers returning from Iraq and Afghanistan and other areas of conflict and is without effective pharmacological therapy. Armed with a generous grant from the Defense Advanced Research Projects Agency, we were able to show that RyR2 channels became leaky in a murine model of PTSD and that either pharmacological (with a Rycal) or genetic (using the non-leaky RyR2-S2808A mouse) treatments could rescue the cognitive and behavioral deficits classically associated with PTSD in this model (26). It was a simple extension of this observation to look at Alzheimer’s disease (AD) using several murine models of familial AD and show that leaky RyR2 channels upstream of amyloid and neurofibrillary tangles could be rescued either pharmacologically (with a Rycal) or genetically by crossing of the mice with RyR2-S2808A mice (27, 28). Most of the therapeutics being tested for AD have targeted some aspect of the amyloid pathway or amyloid itself, but without success. Targeting of Ca^2+^ has been thought of for decades (84), and our data and those of others (85) provide fresh impetus to take another look at this possibility.

## Conclusions

The power of structural biological studies to unveil new understandings of fundamental physiology and pathophysiology is revolutionizing the ways in which we can think about human disorders and therapeutics. Combined with genomic studies and physiological measurements, high-resolution structural studies of large proteins and complexes in multiple configurations that have become possible using cryo-EM are functionalizing the genome in unprecedented ways. The RyR story is a great example of the power of these approaches. Work spanning 35 years led to cloning of the channel, which directly led to its identification as the key Ca^2+^ release channel required for excitation-contraction coupling in muscles, to the discovery of its role in the pathogenesis of human disease including HF and cardiac arrhythmias, to drugging of the channel, and now to elucidation of the structural bases for the pathological leak and the mechanism of action of the Rycal drugs to fix the leak. With each new discovery additional doors are opened, and new questions appear that were both impossible to ask and unanswerable in recent years.

The current strategy is to focus on rare inherited disorders caused by mutations in RyR1 or RyR2 in which Rycal drugs could be disease modifying as a proof of concept for targeting RyR channels to treat human diseases. If these studies show promise, they should open the way for testing Rycals in more common diseases in which there is evidence for RyR channel leak as a major contributing factor.

## Version 1 01/17/2023

Electronic publication
